# Tolerance of Opium

**Published:** 1849-01

**Authors:** 


*Tolerance of Opium.*—Mr. Godfrey, (of Bristol,) narrated two cases at present under his care, illustrative of the large doses of opium that can be borne without narcotism. In one the lady swallowed frequently forty grains of opium within the day; in the other, the patient who for years had been subject to violent neurotic attacks, often took within the day sixty grains of the acetate of morphia.

Mr. Norman some years ago had a gentleman who suffered from senile gangrene, from which he recovered ; he then took opium, and subsequently took a wineglassful of laudanum regularly twice a-day. He obtained from Apothecaries' Hall twenty drachms of opium, which after cutting up he macerated for above a month in a quart of brandy; of this tincture he took two glasses a-day, without any further sensible effect than to exhilarate his spirits. While taking this, on two occasions constipation came on, with imminent risk to his life. After the second attack, he at once left off all his opium, and lived four or five years afterwards, dying eventually of disease of the brain.

Dr. Blackmore had known four hundred grains taken in one day, without narcotism being produced.—*Prov. Med. and Surg. Journ.*

